# Progress of Medical Science—Selections from Foreign Journals—On the Treatment of Croup

**Published:** 1858-11

**Authors:** 


*Progress of Medical Science—Selections from Foreign Journals— On the Treatmeut of Croup.* By Dr. Luzinsky.

Dr. Luzinsky, medical director of the children's hospital at Vienna, after animadverting upon the discrepancy of opinion which prevails concerning the nature and treatment of croup, observes that it is essentially an inflammatory affection of the mucous membrane of the air passages. Still, it is not a mere local affection of these, in as much as croupy exudations are also often found upon other parts of the external or internal surface; so that it arises from a peculiar eondition or *crasis* of the blood giving rise to pseudo-membranous deposits, and is a mere local expression of a general diathesis. The spasm which plays so importaat a part in the disease, is a secondary condition excited by this inflammation.

Speaking of the symptoms, the author observes that the true croupy tone, bearing the most striking resemblance to the barking of a young dog, once heard can never be forgotten. Some practitioners state that this peculiar cough is proper to certain children in every catarrh, and Mauthner says that the cough in short-necked children easily takes on this croupy character in simple broncbial catarrh, as also in children suffering from worms. These statements, Dr. Luzinsky has never been able to verify during fifteen years' observation of many thousand children. The warning given by the child's hoarseness and croupy tone is too often overlooked by friends and practitioners, or many more children might be saved.

The following are the indications of treatment. 1. *To act upon the peculiar crasis of the blood.* This is indeed unconsciously done by almost all the means that have obtained renown, as they all agr[illegible]e in the final effects of diminishing the plasticity of the blood. This is the case with mercurials, antimonials; sulphate of copper, and liver of sulphur. The influence of these latter articles, however, as solvents and alteratives, is too feeble, while their emetic properties in the early stages of croup produce mischievous concussion of the brain and larynx. Mercurials have the disadvantage of exciting diarrhœa or salivation, and when employed to a sufficient extent to operate upon the crasis, may themselves produce a mischievous contamination of that fluid,—a mercurialismus. Alkalies, on the other hand, without being accompanied with any of the above disadvantages, possess great fluidifying power over albuminous and fibrinous products. They have been long employed in diseases depending upon a hyperplastic condition of the blood, and were recommended strongly by some of the older writers in pleuropneumonia and croup. Dr. Luzinsky published in 1855 and 1856 an account of the excellent results that followed his employment of carbonate of potash; and since then, these results have been con firmed by other French and German practitioners. Nevertheless, the old practice of leeching is still continued by the great bulk, and is followed by a mortality of from 60 to 70 per cent. Caustic potash is the form of alkali which of all others most strongly possesses the antiplastic power, but even in the highest state of dilution it proves too irritasing to the digestive organs. As a carbonate, it is far milder in its operation, and can be administered in large and long-continued doses. When continued too long, diarrhœa and excessive fluidity of the blood are the results if it be not suspended. Soda is milder both in taste and influence; and the bicarbonates are only suitable for slight cases. The doses of the carbonate of potass or soda are from ½ to ʒii per diem dissolved in water and sweetened with syrup. The alkali is continued until the cough becomes easier and looser, when usually a plastic, puriform mucus is expectorated.

2. *To prevent the localization of the inflammation in the larynx.*— For this purpose it has been the habit to apply leeches. How little was to be expected from this may be judged of from the extremely slight connection that exists between the blood vessels of the skin and those of the mucous membrane of the larynx. The author regards depletion of any kind as injurious; and at the recent meeting of the German Physicians at Bonn, the younger practitioners were found to hold the same views. Cold would seem *a priori* the best means for preventing or suppressing this inflammation; and Lauda has frequently found the hydropathic application of this successful. Dr. Luzinsky also, in former years, frequently resorted to this, which, however, requires great precautions in respect to the accompanying diseases, the individual constitution of the patient, and the prejudices of the friends. The best means of applying cold is by keeping the base of the neck, as low down as the sternum, covered with cloths wetted in cold, and afterwards in iced water, keeping the rest of the body warm, and assiduously administering small quantities of cold milk and water. This practice is to be continued for from one to three days, when the cough becomes looser and the respiration easier. The cold is then to be gradually decreased, and more nourishment administered. Warm cataplasms are very objectionable, encouraging determination of blood to the parts affected, obstructing the breathing, or rendering the child liable to chills.

Reasonable and useful as is the application of cold, invincible obstacles are sometimes offered to it, and we must then resort to derivative applications in the form of blisters, placed just above the sternum. To this means the author attaches great importance. The surface of the blister soon becomes covered with diphtheritic membranes. When these appear and have frequently to be removed, it is a good sign; while when they are absent, and the secretions from the blistered surface defective, the child is usually lost.

3. *To relieve the spasm of the Laryux.*—For the relief of the spasmodic symptoms exhibited by the distressing cough, the impending suffocation, and the great restlessness, opiates are of great service. They are so especially at the commencement, by relieving the severe cough and procuring repose for the larynx.

4. *The destruction or expulsion of the pseudo-membranes.*—The destruction of the pseudo-membranes has been attempted by means of various caustics; and of all these the author has found the nitrate of silver of the greatest service. A solution of 8 to 16 grains to the ounce should be carefully applied by means of a pencil, several times a day, as low down towards the larynx as possible. When the false membranes begin to loosen and separate in the larynx, or when it is filled with plastic mucus or puriform membrane, and the child, unable to expectorate, is threatened with suffocation, emetics, which at an early stage can only do harm, are now indicated; of these the author prefers the cupri sulph., giving from 2 to 8 grains in 2 ounces of water and 1 ounce of syrup, in teaspoonful doses, every quarter or half-hour, until vomiting is produced. If the carbonate of potass has been employed at an earlier stage, the vomiting will usually cause the discharge of plastic puriform mucus, or, more rarely, of pseudo-membranes.

A child treated according to these indications, freed from its croup, requires little after-treatment beyond diet; and recovers much more rapidly than when he has been weakened by bleeding, emetics and purgatives, and his juices have become impregnated with mercury. As to tracheotomy, the author has no personal experience, and evidently he does not think well of it.

While during three years among 15,000 cases of children's diseases Dr. Luzinsky met with but 30 cases of croup; in another year he met with 60 cases among 23,000 patients. This he attributes to the reputation his successful treatment of the disease had acquired for him. Of these 90 cases, 55 occurred in boys, and 35 in girls, and the ages were as follow:—Under 1 year, 11 cases; from 1 to 2, 16; from 2 to

3, 16; from 3 to 4, 8; from 5 to 6, 15; from 6 to 7, 14; and 9 years old, 1. With respect to the severity of the symptoms, these cases may be divided into three groups. The *first* is characterized by a hoarse voice, by a short, rough, barking cough, difficult respiration, indistinctness of respiratory murmur, with occasional whistling, more or less fever, and constancy of symptoms. The 36 cases of this group all recovered, because the disease was energetically attacked from the commencement. It is a class of cases calling especially for attention, because it often follows measles and influenza. Great care was taken in ascertaining that these were cases of true croup; and that every case exhibiting hoarseness with a rough cough and fever was not so designated, is shown by the fact that 100 cases were left unnoticed, under the designation of catarrh of the larynx. The *second* group is distinguished by a weak, thin, screeching voice, a soundless, tubular cough, labored respiration, a very feeble and hissing respiratory murmur, and great restlessness. In 43 patients the narrowing of the larynx had reached this point. It comprehended partly cases in which the early symptoms had been disregarded, or had not yielded to the means employed, and partly those in which the most suitable means promptly applied, failed to check the progress of the disease. Of these cases 9 died and 34 recovered. In 5 of these the friends utterly neglected the means advised. The *third* group is marked by loss of voice, mere whispering remaining, a dry, suffocating, scarcely audible cough, and a high degree of orthopnea, the respiratory murmur being inaudible, and strangulation seeming imminent. In this condition were 11 cases, of which 5 recovered and 6 died. The disease had proceeded with such rapidity as to leave no time for the operation of remedies, or the pseudo-membranes had already formed when the children were brought in. In three of the recoveries suffocation seemed imminent, and the children were only saved by the active employment of the nitrate of silver.

As the general result of the 90 cases, 75 recovered and 15 died. In the first stage of the disease, all the 36 recovered; in the second, 34 of 43; and in the third, 5 of 11. The conclusion to be drawu is that the treatment should be prompt and active, before the crasis of the blood has become completely developed, and the inflammation localized.—*Journal für Kinderkrankheiten.* Band xxix. pp. 159—176.

[In a subsequent number of the Journal (B. xxx. p. 209), Dr. Hauner, Director of the Children's Hospital at Munich, enters into a criticism upon Dr. Luzinsky's opinions, and concluded his paper with the following aphorisms, which, in a forthcoming work, he intends developing at full length. 1. True croup (laryngeal croup), is a disease proper to childhood, and its cause is chiefly to be sought in the organization (the period of development) of the larynx at this period of life. 2. The anatomy and physiology of the larynx sufficiently explain the nature of croup. It cannot be shown that croup is connected with any peculiarity of the blood-crasis. 4. True croup always commences in the larynx, and often passes downwards to the trachea, etc.; but it never passes upwards. 5. Laryngeal croup is characterized by a pseudo-membrane of more or less extent. 6. Laryngeal croup is to be carefully distinguished from diphtheritic croup, the latter always depending upon a peculiar blood-crasis, as seen in other organs of enfeebled individuals. 7. Diphtheritic croup is almost always secondary, and is not essentially different from croup in and after acute exanthemata. 8. The diphtheritic form begins as a general rule in the fauces, uvula, tonsils, etc., and extends hence downwards. It is very rare for it to commence in the larynx or trachea. A laryngeal catarrh may simulate laryngeal and diphtheritic croup very closely, but in it there is no formation of pseudo-membrane. 10. Such cases are very frequently mistaken for true cronp. 11. There is no specific remedy in true croup, the treatment having to be adapted to the individual cases. 12. Emetics, cold, blood-letting, mercury, etc., are the means, adapted to special cases, that must be relied upon. 13. In certain cases of true croup an operation is desirable. 14. Diphtheritic croup requires for its treatment cauterization, emetics, alkalies, and corroborants; but calomel, bleeding, blisters, or purgatives should never be employed. 15. Tracheotomy is seldom advisable, especially on account of the liability to return of the diphtheritic process. 16. When performed it must be followed by cauterization. 17. The severest laryngeal catarrh yields, as a general rule, to antiphlogistics and suitable regimen. The favorable results obtained in many such cases have been set down as examples of the cure of croup.—*London Medical Times and Gazette.*

